# Ready, set, go!: exploring use of a readiness process to implement pharmacy services

**DOI:** 10.1186/s43058-020-00036-2

**Published:** 2020-06-10

**Authors:** Melanie Livet, Mary Yannayon, Chloe Richard, Lindsay Sorge, Paul Scanlon

**Affiliations:** 1 Center for Medication Optimization (CMO), Division of Practice Advancement and Clinical Education, Eshelman School of Pharmacy, University of North Carolina at Chapel Hill, Chapel Hill, NC USA; 2 Alliance for Integrated Medication Management, Minneapolis, MN USA; 3 Department of Pharmaceutical Care and Health Systems, College of Pharmacy, University of Minnesota, Minneapolis, MN USA; 4 Financial Transformations Inc., Fairfax, VA USA

**Keywords:** Implementation readiness, Readiness building strategies, Readiness facilitators, Readiness barriers, Use of a readiness process, Pharmacy, Medication management services

## Abstract

**Background:**

Readiness is an essential precursor of successful implementation; however, its conceptualization and application has proved elusive. R = MC^2^ operationalizes readiness for use in practice. The purpose of this study was to (1) describe the application of R = MC^2^ to assess and build readiness in nine healthcare sites responsible for implementing medication management services and (2) gain insights into the sites’ experience.

**Methods:**

This mixed methods exploratory study used data collected as part of a process evaluation. Understanding application of the readiness process (Aim 1) involved examining team members’ involvement (who?), readiness challenges and readiness building strategies (what?), strategy execution (how much?), and resulting changes (for what purpose?). To understand the sites’ experience with the R = MC^2^ system (Aim 2), interviews were conducted with six of the sites to identify facilitators, barriers, and lessons learned. Data sources included a document review (e.g., sites’ action plans), survey results, and interview data.

**Results:**

Sites included primary care and specialty clinics, pharmacies within health systems, and community pharmacies. Teams consisted of 4–11 members, including a lead pharmacist. The teams’ readiness activities clustered into five broad categories of readiness building strategies (e.g., building the operational infrastructure for service integration). Of the 34 strategies identified across sites, 68% were still in progress after 4 months. Engaging in the readiness process resulted in a number of outputs (e.g., data management systems) and benefits (e.g., an opportunity to ensure alignment of priorities and fit of the intervention). Based on the interviews, facilitators of the readiness process included assistance from a coach, internal support, and access to the readiness tools. Competing priorities and lack of resources, timely decision-making, and the timing of the readiness process were cited as barriers. The importance of service fit, stakeholder engagement, access to a structured approach, and rightsizing the readiness process emerged as lessons learned.

**Conclusions:**

These findings provide valuable insights into the application of a readiness process. If readiness is to be integrated into routine practice as part of any implementation effort, it is critical to gain a better understanding of its application and value.

Contributions to the literature

- Although implementation readiness (i.e., motivation and capacity) is an essential precursor of successful implementation, its conceptualization has proved elusive. R = MC^2^ operationalizes readiness for use in practice, yet its use and value remain unexplored.
- This is the first published study to systematically explore application of the R = MC^2^ process, with the purpose being to better understand its use in real-world settings.
- This study addresses a recognized gap in the implementation science literature, contributing valuable insights into use of a readiness process. These findings add to the current body of literature by identifying lessons learned that should be considered when building implementation readiness.

## Introduction

Implementation readiness has been recognized as an essential precursor of successful implementation [1–6]. A practice setting needs to both be willing and able to carry out a change [1, 6–8], whether this change is implementation of a new service, intervention, technology, or policy. Assessing and building readiness has oftentimes been overlooked by well-meaning stakeholders eager to jump into action. Discounting readiness can be costly, resulting in avoidable implementation misadventures and, subsequently, failure to achieve the intended outcomes [6, 9]. In fact, failure to establish sufficient readiness prior to implementation accounts for half of all unsuccessful, large-scale organizational change efforts [10]. Readiness should be part of any translational efforts and has been identified as a critical step in implementation practice roadmaps [11] and frameworks [2, 12–14].

Despite the general consensus that readiness is an important aspect of implementation, its conceptualization and application in real-world settings has proved rather elusive [2, 5–7, 12, 15, 16]. Although organizational change models and theories abound [17–19], very few frameworks operationalize the readiness process for use in practice [20]. One of these frameworks is R = MC^2^ [20]. Briefly, R = MC^2^ defines readiness as a multi-faceted construct that refers to an organization’s commitment and collective capability for implementation. It posits that readiness results from the interplay between three components: motivation (i.e., incentives and disincentives that contribute to the desirability of an innovation), general capacity (i.e., conditions related to how well an organization is functioning), and innovation-specific capacity (i.e., conditions needed to implement a specific innovation). The more willing and able an organization is to devote the resources necessary, the greater the likelihood of quality implementation [2, 4–6, 21]. Each of these components is further conceptualized into multiple subcomponents (see Table 1). This heuristic was translated into an assessment and readiness building system that includes practical tools and specific strategies to facilitate execution. The R = MC^2^ system was used as the readiness framework for this study.

**Table 1:** Readiness components and subcomponents

**General capacity**	**The overall functioning of an organization.**
Culture	Expectations, norms, and values of how things are done in this organization.
Climate	Employees’ perceptions, appraisals, and feel about their current working environment.
Structure	Processes that impact how well a site functions on a day-to-day basis.
Organizational innovativeness	Openness to change at this organization.
Resource utilization	Ability to acquire and allocate resources including time, money, effort, and technology.
Leadership	How effectively management sets tone and expectations at this organization.
Staff capacity	The number, experience, and skill level of individuals at this organization.
**Innovation-specific capacity**	**What is needed to make the intervention happen.**
Innovation-specific knowledge, skills, and abilities	Knowledge, skills, and abilities required to implement the intervention with quality.
Program champion	A well-connected person(s) who supports, promotes, and puts his or her influence behind the intervention.
Supportive climate	Necessary supports, processes, and resources needed to implement the intervention.
Inter-organizational relationships	Relationships with others outside of the organization that facilitate use of the intervention.
**Motivation**	**Degree to which individuals within the organization want the intervention to happen.**
Relative advantage	Degree to which the intervention seems to be advantageous for this site.
Compatibility	Extent to which the intervention fits with the existing cultural values, needs, and current practices
Complexity	Degree to which the intervention can be implemented with ease.
Observability	Extent to which the small wins from using the intervention are visible to others.
Priority	Importance of the intervention compared to other demands.
Ability to pilot (trialability)	Degree to which the intervention can be tested and experimented with.

From Scaccia et al. [20]

In addition to the sparsity of pragmatic readiness frameworks, the published literature on how to apply these frameworks is in its infancy. To date, there are only two published examples illustrating the application of the R = MC^2^. The first study uses the R = MC^2^ readiness monitoring tool (RMT) to monitor changes in five schools’ readiness to implement a school safety intervention [22]. In the second article, the authors conducted a Delphi study with community coalition leaders to assess the relative significance of each of the R = MC^2^ concepts at different stages of the implementation process [23]. The current study adds to the literature by exploring application of R = MC^2^ in healthcare settings. More specifically, R = MC^2^ was used to assess and build readiness for implementing medication optimization services as part of a broader pharmacy practice initiative, the Concordia Medication Management Accelerator (CMMA). Briefly, CMMA aimed to accelerate integration of clinical pharmacy services in participating healthcare settings through a structured planning approach that included a readiness phase.

 The aims of this mixed methods exploratory study were to (1) describe the application of the process to assess and build readiness in an initial cohort of nine healthcare sites, including seven health systems/clinics (with embedded pharmacists) and two community pharmacies, and (2) understand the experience of participating sites with the readiness process. If readiness is to be integrated into routine practice as part of any implementation effort, it is critical to gain insights into its application and value.

## Methods

### Overview of study design

This mixed methods exploratory study made use of data collected as part of a process evaluation to better understand the use of the readiness process, as well as the sites’ experience with it. The description of the R = MC^2^ system as applied to practice (Aim 1) involves understanding the participating teams and their involvement (who?), examining the types of readiness challenges and readiness building strategies used by the sites (what?), summarizing progress on strategy execution (how much?), and investigating resulting changes (for what purpose?). Aim 1 was informed by multiple data sources, including project documents, surveys, and relevant information from site interviews. To understand the sites’ experience with the R = MC^2^ system (Aim 2), interviews were conducted with six of the sites to identify facilitators, barriers, and lessons learned. Following IRB approval, Aim 1 data were collected throughout the 9-month readiness phase. Aim 2 interviews occurred at the end of the readiness phase.

### Readiness in pharmacy practice: the CMMA initiative

The importance of attending to the implementation process has recently been embraced by pharmacy practice as a potential solution to accelerate the pace of healthcare change, drive effectiveness of medication optimization interventions, and facilitate replication and scaling [24, 25]. Suboptimal use of medication not only impacts quality of care, but it is also one of the most preventable problems contributing to rising healthcare costs (e.g., $528 billion annually spent on addressing medication misuse vs $450 billion spent on prescriptions) [26, 27]. Interest in the process of implementation has been fueled by both the transition to value-based care and the lack of conclusive effectiveness outcomes in the medication optimization literature [25, 28, 29]. Reducing implementation variability through planned and systematic delivery of services begins with assessing and building readiness.

The Concordia Medication Management Accelerator (CMMA) was an 18-month initiative to integrate medication optimization services into primary care settings across the health system in Wisconsin. This initiative was sponsored by the Concordia University Wisconsin’s School of Pharmacy in partnership with the Batterman School of Business. Funding for this initiative was a personal gift from Erv Dohmen. The nine participating sites, composed of seven health systems/clinics (with embedded pharmacists) and two community pharmacies, were recruited through an open invitation to participate in a live summer event at Concordia University Wisconsin. Any healthcare organization, primary care clinic, health system, and pharmacy across Wisconsin working to advance or adopt medication optimization services within their organization was eligible to participate. Over the course of this project, the sites engaged in a structured planning and implementation process that included readiness as one of its steps. Implementation support, including monthly coaching, webinars, and in-person meetings, were provided to the sites by an external non-profit organization, the Alliance for Integrated Medication Management (AIMM). AIMM works to drive changes in care delivery systems [30]. The readiness project team, composed of two AIMM coaches and the UNC Eshelman School of Pharmacy Center for Medication Optimization (CMO) research group, was specifically dedicated to assessing and building readiness over a 9-month period.

### Operationalizing R = MC^2^ in the CMMA initiative

The R = MC^2^ process was operationalized into four phases: preparing for readiness (1 month); readiness assessment (3 months); priority identification, goal setting, and action planning (1 month); and strategy execution (4 months).

#### Preparing for readiness

Prior to engaging in the readiness work, each participating site was asked to identify a lead pharmacist whose first responsibility was to assemble an implementation team. These teams were responsible for planning and implementing the selected service(s), starting with assessing and building readiness. Once the teams were formed, the concept of readiness, and more specifically R = MC^2^, was introduced during two webinars. These webinars were recorded for team members who were unable to attend. In addition, coaches had preliminary discussions with each of the teams to clarify the purpose of the readiness process, explain how this process was integrated into the broader initiative, and address any concerns that were raised by the teams.

#### Assessing readiness

During the readiness assessment phase, each team completed the RMT. To assist the teams with use of the RMT data, the CMO research group created individual heatmap reports. These reports provided reminder information about readiness, RMT assessment results specific to the team, a list of strengths, challenges, and key insights, and a summary designed to assist with selection of priority readiness building areas. The heatmap reports were shared with teams via email and discussed during their regularly scheduled coaching check-ins. These collaborative discussions were not only necessary for the teams to understand their results, but proved to be critical to help guide priority setting.

#### Identifying priorities, setting goals, and action planning

Following these discussions, the teams prioritized their readiness challenges on feasibility and impact (high or low) using a four-quadrant priority matrix. These priorities were then translated into actionable readiness goals with assistance from the coaches. Simultaneously, the CMO research group assembled a preliminary list of relevant readiness building strategies for each team based on the RMT results and priorities. At least one strategy was identified for each RMT item that was recognized as a priority issue for readiness building. The recommended strategies were shared with the coaches and teams to facilitate concrete discussions for action planning. Ultimately, the teams decided on the most relevant strategies for their action plan.

#### Readiness building strategies execution

Teams were asked to execute on their selected readiness building strategies over a 4-month period. During this period, coaches checked in monthly and used the action plan to monitor progress and facilitate discussions around challenges and potential solutions.

### Data collection procedures and data sources

**Fig. 1:**
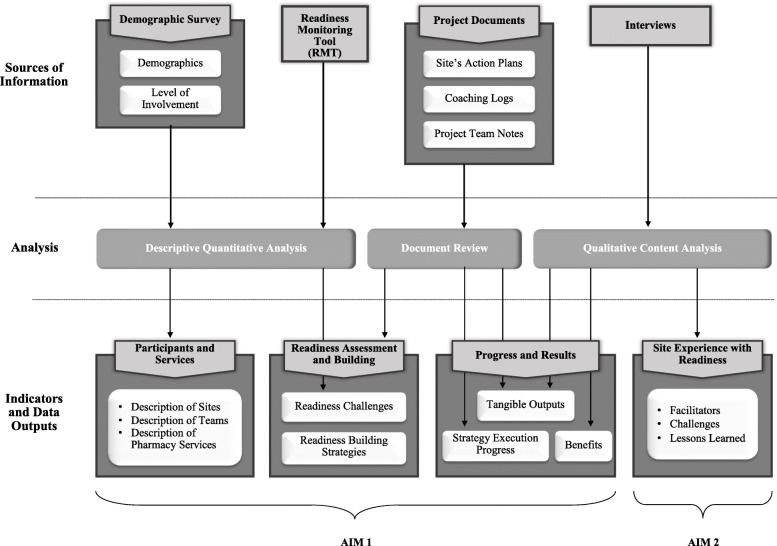
Overview of data sources, analysis, and resulting data outputs

Aim 1 data sources consisted of the following: project documents (i.e., the sites’ readiness action plans, coaching logs, and the readiness project team notes); two surveys, including a demographics survey that was distributed to each team member at each site, and the RMT that was completed by each team as part of the readiness process; and Aim 2 interview information specifically related tochanges made as a result of engaging in the readiness process. Aim 2 data were generated through interviews with six lead pharmacists, one from each site. Although the invitation to interview was extended to all the participating sites, two of the lead pharmacists were unable to participate due to time constraints. Participant consent was obtained prior to both surveys and interviews. Figure 1 provides an overview of data sources, analysis, and resulting data outputs.

#### Surveys

The *demographics survey* was completed by team members at the end of the project. In addition to demographic questions (e.g., gender, age, ethnicity, race, highest degree completed, professional role), the survey for the lead pharmacist included items inquiring about the level of involvement of each team member in the readiness process (i.e., completing the readiness assessment, reviewing the report and identifying priorities, developing the action plan, executing on the readiness building strategies, and reviewing progress of readiness actions). Ratings ranged from “not at all involved” to “fully involved” on a 3-point scale.

The *RMT* used in this study was a 67-item Likert-scale created to assess level of readiness for implementation. It is composed of three scales, aligned with the three components that conceptually define readiness (i.e., motivation, general capacity, innovation-specific capacity). Each of the scales is further divided into subscales representing the 17 subcomponents (e.g., culture, program champion) (see Table 1). Items (e.g., the team has the concrete skills needed to implement the selected service) are scored on a 7-point Likert scale, ranging from strongly disagree to strongly agree with a neutral option. Minor wording changes were made to the original RMT [31] to reflect the study context (e.g., “organization” to “site”). The original RMT is available from its developers at the Wandersman Center. This measure was shown to have good reliability (Cronbach’s alpha; α ≥ 0.070 for 89% of the subcomponent index scores) [31]. In addition to rating the RMT Likert-type items, the team was also asked to generate their top three readiness insights.

#### Project documents

The data sources for the document review included the *sites’ readiness action plans*, the *coaching logs*, and the *readiness* *project team notes*. Action plans were completed by each site as part of the readiness process. In these plans, the sites documented their readiness priorities/goals, their readiness building strategies, associated tasks, the team member(s) responsible for completing these tasks, due dates, and progress to date. The coaching logs were completed by the coaches after each site’s check-in to document readiness-related discussions. Although the coaching logs included additional information (e.g., date of contact, coaching recipient, coach name, duration of the coaching session), the discussion notes were the only data used in the current study. Finally, the project team notes included both summaries of conversations among the readiness project team members (research team and coaches) (*N* = 9) and the notes taken by the research team members when able to attend the coaching calls (*N* = 16).

#### Interviews

An interview protocol was created to standardize the semi-structured interviews. The interview questions, which were provided to the participants ahead of time, were designed to identify what worked well with the readiness process, the challenges that were encountered, the changes that were experienced as a result of the readiness building process, and lessons learned. While these questions helped define the areas to be explored, the interviewer and interviewee were allowed to diverge to pursue an idea in more detail [32]. To ensure that they had a common frame of reference when responding to these questions, participants were reminded of the specific activities that were part of the readiness process (e.g., completing the readiness assessment, identifying priorities). They were also provided with a summary of these activities for their specific team (e.g., one of your priority readiness goals was to obtain buy-in for comprehensive medication management (CMM) from your office manager). These 30-min interviews were conducted over a 2-week period by a trained interviewer. They were transcribed verbatim to facilitate analysis following consent from each participant.

### Data analysis

#### Surveys

Descriptive statistics (frequencies, means) were calculated across respondents on the demographics survey to obtain a description of the participants across teams. In addition, the level of involvement items in the demographics survey were averaged across readiness activities and participants for each team, resulting in percent involvement. Finally, RMT survey scores were created by averaging survey items across teams. Means, standard deviations, and frequencies were computed by components and subcomponents. Components and subcomponents with a mean rating equal or above 4.5 represented high levels of readiness, between 3.5 and 4.49 as moderate, and below 3.5 as low.

#### Document review

The document review was used as a methodology to provide data on application of the readiness system [33]. As for other types of qualitative analyses, document analysis involves review of the information from the different data sources, abstraction into categories of interest, and interpretation to elicit meaning and understanding. Information from all data sources (coaching logs, action plans, and notes) was first compiled for each site separately in a log that ordered these data chronologically. These site logs were then reviewed and abstracted into site-specific spreadsheets by two members of the CMO research team using the following categories: priority/goal, RMT components/subcomponents, readiness building strategies, and progress to date. The CMO research team then reviewed, interpreted, and standardized this information through discussion and consensus building [34]. These data were aggregated across sites to summarize the sites’ priorities, readiness building strategies, and progress.

#### Interviews

Interview transcripts were analyzed using content analysis [35]. During the pre-coding stage, the analyst read the transcripts to become familiar with the material. Primary codes were then applied to each relevant line of text, with secondary codes emerging as a result of a second read. Primary codes included successes/facilitators, challenges, changes, and lessons learned. The codebook was refined to include primary and secondary codes. The third read involved focused coding of the transcripts. The information was synthesized into a spreadsheet that included the codes, subcodes, and relevant transcript text for each site, allowing for comparisons across sites and codes. The analysis was conducted by the second author, with quality assurance checks performed by the third author on 20% of the interviews. Kappa coefficients demonstrate agreement between analysts, with a Cohen’s kappa of 0.88.

## Results

### Understanding use of the readiness process (Aim 1)

#### Sites, teams, and pharmacy services

**Fig. 2:**
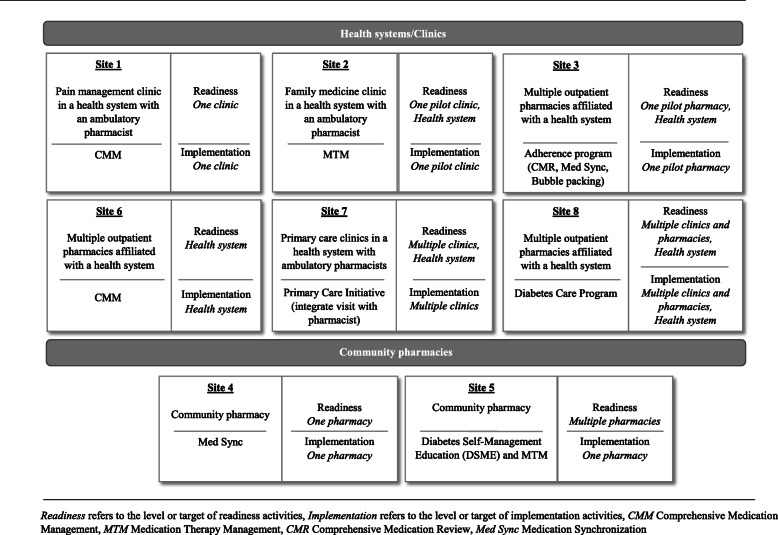
Description of sites

All but one site (that dropped out of the broader initiative due to changes in priorities) engaged in the readiness assessment and building activities. This convenience sample included six health systems/clinics and two community pharmacies. Figure 2 provides a brief description of each site. Sites included primary care and specialty clinics within health systems, outpatient pharmacies within health systems, and community pharmacies. The medication optimization services and projects that were selected for implementation were unique to the needs of each site. Examples included comprehensive medication management (CMM), medication therapy management (MTM), medication synchronization (Med Sync), multi-faceted medication adherence programs (e.g., comprehensive medication reviews (CMRs), bubble packing), and diabetes management. In addition, because each site differed in the intended scope of implementation (e.g., one vs. multiple settings), the levels at which readiness needed to be built was also varied. While some sites focused on readiness building in one setting, others selected to build readiness in multiple settings and/or at the health system level (Fig. 2).

Each implementation team included between 4 and 11 members, including at least one pharmacist, medical professionals (e.g., nurse practitioner, physician), administrators and support staff (e.g., operations manager), patient advocates and educators, behavioral health (e.g., psychologist), and other relevant stakeholders (e.g., dietitian) (Table 2). When examining the level of involvement of each team member (reported on the demographics survey), it is worth noting that 1–3 individuals per team (rather than the full team) were fully engaged with completion of all of the readiness assessment and building activities (Table 2). A brief description of each team's intervention focus is also included in Table 2.

**Table 2:** Description of intervention focus and teams

Site	Intervention Focus	Team members		
		N	Role (*N* if > 1)	Involvement (*N*)
1	Implement CMM and position pharmacist as a physician extender	9	Clinical Pharmacist* Clinical Pharmacy Manager Psychologist Physicians (2) Physician’s Assistant Medical Assistants (3)	11% fully involved (1) 89% not involved (8)
2	Implement MTM to reduce heart failure admissions and improve diabetes care	8	Clinical Pharmacy Manager* Clinical Pharmacy Resident* Operations Manager Operations Supervisor Nurse Practitioner Physicians (3)	25% fully involved (2) 75% not involved (6)
3	Combine CMRs, bubble packing, and Med Sync into a comprehensive adherence program	9	Clinical Pharmacy Coordinators(2)* Clinical Pharmacists (3) Pharmacy Services Director Pharmacy Manager Pharmacy Resident Physician	22% fully involved (2) 78% not involved (7)
4	Implement Med Sync to increase patient adherence	4	Pharmacy Resident* Clinical Pharmacist Patient Care Advocate Patient Care Advocate Assistant	25% fully involved (1) 75% not involved (3)
5	Expand the diabetes self-management education program to include MTM	4	Clinical Pharmacist* (2) Pharmacist* Pharmacy Technician	50% fully involved (2) 50% not involved (2)
6	Implement CMM to reduce the overall cost for high-risk employee patients	8	Pharmacy Director* (2) Pharmacy Manager (2) Pharmacists (3) Pharmacy Resident	13% fully involved (1) 26% somewhat involved (2) 61% not involved (5)
7	Incorporate a follow-up pharmacotherapist visit for patients with chronic conditions	10	Pharmacy Resident* Pharmacy Manager* Pharmacists (3) Physicians* (3) Data Analyst Marketing	33% fully involved (3) 70% not involved (7)
8	Develop a collaborative coordinated diabetes care program to reduce overall cost for high-cost employee patients	11	Director of Pharmacy* Physician Clinic Nurse Manager Pharmacist Manager (2) RN Diabetes Educator* Health and Fitness Coordinator Dietitian Program Educator* (2) Rehab Director	27% fully involved* (3) 55% somewhat involved (6) 18% not involved (2)

*CMM* comprehensive medication management, *MTM* medication therapy management, *CMR* comprehensive medication review, *Med Sync* medication synchronization

*Denotes “fully involved”

#### Readiness challenges and readiness building strategies

Baseline RMT scores for all of the sites were high across readiness components and subcomponents, with means above 4.5 (Table 3). Based on an item-level analysis average across the sites, there seems to be uncertainty around the ease of implementing the intervention (i.e., the team believes that the selected intervention is easy to implement, *M* = 3.89) and resource allocation (i.e., there is a clear process by which we prioritize and distribute resources, *M* = 4.33). Based on the document review, the coaches’ discussions with each of the sites about their assessment results uncovered additional readiness challenges. The compiled list of prioritized challenges is presented in Table 4. Operational integration of the service and stakeholder engagement/buy-in were selected as priority challenges by the majority of the sites (all but one site, and five of the eight sites, respectively). The readiness building strategies used by the sites were aligned with their priority challenges. Table 4 provides a list of strategies used by the sites, as well as specific examples.

**Table 3:** Readiness scores by component and subcomponent (across sites)

Component and subcomponent	Sites			
	Mean (SD)	Frequencies (*N*)		
	(*N* = 8)	High scores, ≥ 4.5	Neutral scores, 3.5 to 4.49	Low scores, < 3.5
**General capacity**	5.75 (0.61)	8		
Culture	6.06 (0.65)	8		
Climate	5.81 (0.76)	8		
Structure	5.78 (0.82)	7	1	
Organizational innovativeness	5.85 (1.02)	7	1	
Resource utilization	4.91 (0.84)	6	2	
Leadership	6.02 (0.6)	8		
Staff capacity	5.79 (0.99)	7	1	
**Innovation-specific capacity**	5.53 (1.03)	7		1
Innovation-specific knowledge, skills, and abilities	5.92 (0.81)	7	1	
Program champion	6.04 (1.03)	7	1	
Implementation supports	5.18 (0.95)	5	3	
Inter-organizational relationships	4.88 (1.92)	6		2
**Motivation**	5.72 (0.34)	8		
Relative advantage	5.46 (1.01)	6	2	
Compatibility	6.34 (0.5)	8		
Complexity	6.71 (1.05)	6	1	1
Observability	5.46 (0.45)	8		
Priority	5.46 (1.07)	6	2	
Ability to pilot	5.5 (0.96)	7	1	

Means denote the level of readiness from 1 (lowest) to 7 (highest)

**Table 4:** Prioritized readiness challenges and readiness building strategies

Prioritized readiness challenges	Readiness building strategies	Site examples	Sites	Stage
Operational integration • Data process and systems • Financial resources • Patient information repository • Referral process • Workflow • Staffing	Build data processes and systems to track and monitor	Build data management process and/or system to monitor patient progress and outcomes (e.g., integration with EPIC)	1, 2, 5, 8	Implementation
	Develop reimbursement/financial plan	Develop a payment/billing methodology	6	Implementation
		Develop a financial plan to sustain the initiative through meetings with leadership and by hiring staff	2	Sustainability
		Develop and present a scalability plan showcasing pilot data to obtain additional financial resources through meetings with leadership	3	Scalability
	Create a patient information repository	Develop a centralized repository for patient information by partnering with IT	8	Implementation
	Develop a referral process	Develop a referral process for service by creating a referral form for primary care physicians	5	Implementation
	Develop integrated workflow	Develop workflow for service (e.g., after reviewing current best practices for service, after completing driver diagram)	2, 4	Implementation
		Create workflow to meet the capacity needed to reach a larger high-risk patient population	6	Scalability
	Optimize staffing to maximize efficiencies	Optimize staffing to maximize efficiencies by transitioning roles and responsibilities to mitigate staff turnover	4	Implementation
		Optimize staffing to maximize efficiencies by modifying roles and responsibilities to allow pharmacists to focus on billable services	5	Implementation
Stakeholder engagement and buy-in	Use pilot data	Use pilot data for gaining approval from partnering health plan for payment model	6	Implementation
		Use of pilot data to increase buy-in from leadership for scaling service	3	Scalability
	Market/create promotional messaging	Create and disseminate marketing materials through multiple avenues of communication (e.g., mailing lists, media outlets) to increase patient and provider engagement in the service	8	Implementation
		Brainstorm promotional messaging for internal marketing materials with pharmacy managers to increase pharmacist engagement	6	Implementation
		Deliver promotional messaging to pharmacists through program champions (i.e., pharmacists on board)	6	Implementation
	Conduct trainings	Develop and conduct trainings for pharmacy residents and clinic staff (e.g., on workflows, data collection)	3, 4	Implementation
	Provide education	Educate clinic staff on service and their role	1	Implementation
Team	Identify team members	Identify team members responsible for readiness and implementation	1	Implementation
	Engage team members	Engage implementation team through online training and showcasing of positive patient stories to obtain buy-in for the selected service	4	Implementation
Champion	Identify champion	Identify champion to promote service through discussion with leadership	1	Implementation
	Leverage champion	Leverage champion to develop a billing pathway and help prepare for implementation	1	Implementation
Priority alignment	Determine priority for service	Determine priority for service by reaching out to new leadership once identified (in midst of merger)	7	Sustainability
	Demonstrate value	Continue to deliver pharmacy service and collect data to showcase value to new leadership once identified (in midst of merger)	7	Sustainability
	Obtain feedback	Obtain feedback and support from leadership on priority for scalability of the service through meetings and presentation of pilot data	3	Scalability
Service fit	Clarify needs	Determine expectations for selecting a service through meetings with leadership	1	Implementation
Support	Access coaching	Engage coach to support readiness process	1	Implementation
	Learn from peers	Communicate with peers outside of the organization to learn about billing pathways from providers implementing this service	1	Implementation

#### Progress on strategy execution and resulting changes

Of the 34 readiness building strategies identified across sites, 23% of strategies (*N* = 8) had been completed, 68% (*N* = 23) were in progress, and 9% (*N* = 3) had not been addressed (Table 5). Three of the eight sites reported completing strategies, while all the sites reported some progress towards execution of the strategies.

**Table 5:** Progress on strategy execution

Site	Readiness strategy used	Progress
1	Build data management system to monitor progress and outcomes (excel spreadsheet, then integration with EPIC)	In progress
	Identify champion to promote service through discussion with leadership	Complete
	Leverage champion to develop a billing pathway in order to bill for the service	In progress
	Leverage champion to help prepare for implementation	Complete
	Identify team members responsible for readiness and implementation	Complete
	Determine expectations for selecting a service through meetings with leadership (CMM or opioid tapers)	Complete
	Educate clinic staff about the service through meetings	In progress
	Educate staff about their role in service delivery through meetings	In progress
	Engage coach to support readiness process	Complete
	Communicate with peers outside of the organization to learn about billing pathways used by providers implementing this service	In progress
2	Build data management system as a way to monitor progress and outcomes by creating template in EPIC to capture and report on data for the service	In progress
	Develop a financial plan to sustain the initiative by engaging with leadership and hiring an FTE	In progress
	Develop workflow for service after reviewing current best practices in heart failure	Complete
3	Use of pilot data to increase buy-in from leadership for scaling service	In progress
	Develop and present a scalability plan showcasing pilot data to obtain additional financial resources through presentations and meetings with leadership	In progress
	Obtain feedback and support from leadership on priority for scalability of the service through meetings and presentation of pilot data	In progress
	Train resident on data collection process through meetings and training materials	Not addressed
4	Engage implementation team through online training and showcasing of positive patient stories to obtain buy-in for the selected service	In progress
	Develop workflow to integrate new service after completing driver diagram	In progress
	Develop training on workflow to get staff confident with service delivery	In progress
	Optimize staffing to maximize efficiencies by transitioning roles and responsibilities to mitigate staff turnover	In progress
5	Develop process for tracking patient data	Not addressed
	Develop a referral process for pharmacy service by creating referral form for primary care physicians	In progress
	Optimize staffing to maximize efficiencies by modifying roles and responsibilities to allow pharmacist to focus on billable services	In progress
6	Use pilot data for gaining approval from partnering health plan for payment model	Complete
	Create workflow to meet the capacity needed to reach a larger high-risk patient population	In progress
	Develop a payment/billing methodology	Complete
	Brainstorm promotional messaging for internal marketing materials with pharmacy managers to increase pharmacist engagement	In progress
	Deliver promotional messaging to pharmacists through program champions (i.e., pharmacists on board)	In progress
7	Determine priority for service by reaching out to new leadership once identified (in midst of merger)	In progress
	Continue to deliver pharmacy service and collect data to showcase value to new leadership once identified (in midst of merger)	In progress
8	Build data management system as a way to monitor patient progress and outcomes by partnering with IT	In progress
	Create and disseminate marketing materials through multiple avenues of communication (e.g., mailing lists, media outlets) to increase patient and provider engagement in the service	Not addressed
	Develop a centralized repository for patient information by partnering with IT	In progress

Changes resulting from engaging in the readiness process fell into two categories: tangible outputs and benefits. Tangible outputs included data management systems; billing pathways, payment methodologies, and reimbursement models for the service; trainings and educational materials; service workflows; staffing plans; referral processes; and patient data repositories. Benefits included increased awareness of readiness challenges; an opportunity to ensure alignment of service priorities across the clinic/health system (e.g., “What the readiness results really showed me is that I need to make sure that this is a priority not just for me, but also for the health system and the providers I work with”); the ability to clarify the service that was most appropriate for existing needs (e.g., “in talking with those stakeholders, it really kind of changed my mind a whole lot. I started looking more at okay, is this more Comprehensive Medication Management that I’m doing, is it more just pain specific medication management that I’m doing, and so I took a step back and looked at the big picture for my health system and then providers”); an opportunity to build a case for the service to facilitate communication, increase buy-in, and promote engagement and action (e.g., “We did put out some targeted communication to our pharmacist team to help demonstrate what we were doing and why we were doing it, and to get further people engaged in participating in the process”); and the ability to organize readiness tasks and track progress, with one site mentioning continuing to use this process in other implementation initiatives.

### Understanding experience of participating sites with the readiness process (Aim 2)

#### Facilitators—what promoted use of the readiness process

Based on the interviews, the elements that facilitated use of the readiness process included having support from a coach, access to a program champion, having an engaged team, and access to the readiness tools. Coaches were reported to be helpful in a number of ways. They assisted with reviewing and understanding the readiness assessment results, facilitated the prioritization process, facilitated planning and execution of the readiness building strategies, provided feedback, reviewed progress, and ensured accountability. Interviewees also commented on the importance of a program champion. The program champion was reported to be leveraged to ensure accountability, influence other stakeholders and help getting readiness tasks accomplished, and connect with leadership about decisions that had an impact on the project (e.g., “our supervisor [champion] helped give us which [health system] person we needed to talk to for the blessing of an FTE”). Having a team who was enthusiastic and motivated, focused on execution, understood priorities, and brainstormed together was also cited as a facilitator (e.g., “the team members were all highly invested…we knew we would not let this fail”). Finally, interviewees highlighted the readiness process and tools as being particularly helpful (e.g., “It made us think and make sure we checked all of the boxes. That we did not miss talking to anyone or considered other alternatives to decisions”). The overall process was useful to help them carefully think through decisions, consider differing viewpoints, and prioritize. Specifically, the RMT data and individualized heatmap reports facilitated identification and framing of readiness issues that needed to be addressed; the priority tool helped organize the readiness issues into meaningful categories; and the action plan served multiple functions, including as a communication tool, a roadmap for readiness building execution and progress tracking, and a strategy to demonstrate the value of pharmacists.

#### Challenges—what impeded use of the readiness process

Competing priorities and lack of resources, difficulties with timely decision-making, team issues, and timing of the readiness process emerged as barriers to using the readiness process. Misalignment of priorities at the administrative level included leadership changes, a merger, and functioning within a large bureaucracy. In addition, use of the readiness process was negatively impacted by time and resource constraints. The pharmacists’ and clinic stakeholders’ responsibilities focused first and foremost on patient care (e.g., “I felt uncomfortable even presenting it [readiness assessment] to anyone else that I worked with in the clinic, because I felt like that would create more of a barrier in terms of adding more work for them”). In addition to competing priorities, simple lack of time (e.g., “we weren’t able to devote as much time into the project”), insufficient staffing, and ease of data access (e.g., “...just things like trying to get data, just even baseline data, was very difficult”) were cited as resource barriers. The ability to obtain buy-in and make timely decisions due to logistical difficulties (e.g., “getting everybody in a room”) and changing priorities (e.g., merger) also influenced completion of readiness activities. Issues within the team, such as turnover and difficulties building a team, emerged as significant hindrances to engaging in the readiness process. Finally, because the readiness process overlapped with implementation of the project at more advanced sites, it was perceived as “being too late” along the project lifecycle.

#### Lessons learned

When asked about the advice they would give to a colleague about to engage in the readiness process, interviewees pointed to a number of lessons learned. First, the need to focus the readiness process on a priority service for the healthcare setting was mentioned as being critical to success. Second, the ability to engage others in the readiness process was key to increase buy-in and ensure consideration of multiple perspectives (e.g., “Through that readiness assessment tool I think we learned that we needed input from IT; we needed input from our quality assurance person; so just having all the disciplines that you really need to build the program [...] would have been beneficial”). In addition, communicating and sharing progress across the clinic was reported to legitimize this readiness work. Third, having access to a structured process was found to be valuable to identify priorities, provide a roadmap for action, monitor progress, and facilitate the development of a step-by-step readiness building approach. However, because the readiness process can be time and resource intensive, it was recommended that it be rightsized to the needs of the initiative (e.g., “I kind of like the whole concept, and I’ve been adapting some of the concepts for smaller projects, just not doing the whole assessment”) and to organizational maturity level (e.g., “I feel like organizations that were more building from the ground up…could benefit”). Interviewees also pointed out the importance of timing. The readiness process is most valuable early in the project lifecycle, prior to implementation.

## Discussion

Readiness is an essential precursor of successful implementation; however, its conceptualization and application have proved elusive. R = MC^2^ operationalizes readiness for use in practice. Yet, its use and value have not been explored. The purpose of this study was to (1) describe application of R = MC^2^ to assess and build readiness in a cohort of nine healthcare sites responsible for implementing medication management services and (2) gain insights into the sites’ experience with this process. To our knowledge, this is the first published study to systematically explore operationalization of the R = MC^2^ system, with the purpose being to better understand its value in real-world settings.

### Insights into use of the readiness process

Use of the readiness process was synthesized by examining who was involved, what readiness challenges and strategies were identified, how much progress was made toward strategy execution, and to what purpose. Involved in the study were primary care and specialty clinics within health systems, outpatient pharmacies within health systems, and community pharmacies. Teams consisted of 4–11 members, including a lead pharmacist, although completion of the readiness process ended up being the responsibility of 1–3 individuals per team. This type of team structure reflects best practice guidance on building effective teams, with select core members accomplishing the work and other stakeholders participating in an advisory or linkage capacity [36]. Although each team had unique sets of readiness challenges, the readiness building strategies selected for execution clustered into five broad categories (i.e., building the operational infrastructure for service integration, identification and leveraging of teams and champions, stakeholder engagement and buy-in, alignment of priorities and service fit, and the need to access external implementation supports). Previous literature identified similar preparation domains for successful implementation [37–40]. Of the 34 strategies identified across sites, 68% were still in progress after 4 months. Readiness building might require additional time, with previous guidance highlighting the need to continue the process throughout the project lifecycle [20, 31, 41]. Determining the essential aspects of readiness for each implementation stage might be helpful to focus readiness activities. Finally, engaging in the readiness work was associated with a number of benefits (e.g., increased awareness of readiness challenges, an opportunity to ensure alignment of priorities and fit of the intervention). These benefits align with previous research on implementation capacity. Anticipating challenges, assessing fit, obtaining buy-in, and adopting a systematic approach to organizing and tracking progress have been identified as critical precursors of successful implementation [42, 43]. The readiness process also resulted in varied operational outputs (e.g., data management systems, service billing methodologies). These outputs are consistent with the infrastructure needed to implement medication optimization services [44]. Assessing and building readiness seemed to therefore facilitate the identification and development of critical processes and infrastructure for service delivery.

### Insights into the sites’ experience with the readiness process

First, *identifying and building internal supports, such as an accountable team and a champion*, were cited as both readiness challenges and facilitators. Although both the team and the champion were reported to help with accountability, they also had different roles. The team served to support the champion's efforts and assist with discrete tasks, while the champion promoted the change and facilitated execution of readiness activities within the care setting. Previous research has identified internal teams and champions as critical precursors of successful planning, implementation, and sustainability of interventions [38, 39, 45–49].

Second, the *need to engage and communicate with others* within the organization (i.e., healthcare providers, administrative staff, leadership) to build buy-in and promote action was mentioned as a readiness challenge and lesson learned. Advocacy, social mobilization, the ability to incorporate multiple perspectives, and the capacity to navigate the political environment of an organization have all been recognized as necessary relational skills to drive change [38, 47, 50]. Getting others within care delivery settings to embrace the readiness process and understand its benefits (i.e., preparing for successful implementation of a service) is no exception.

Third, *attending to priorities* was emphasized as both a lesson learned and a potential barrier. Engaging in readiness was reported to facilitate alignment of service priorities for the clinic and health system. However, more pressing demands often limited the amount of time and resources that could be allocated to this process. This highlights the needs and resourcing tensions that exist in busy healthcare practices with competing responsibilities.

Finally, participating sites emphasized the value of having *access to a methodical approach and external supports* (i.e., coaching, tools) to be able to effectively use the readiness process. These findings are aligned with previous literature emphasizing the need for active capacity building strategies along the broad implementation continuum (from planning to sustainability) [51–53]. Participants also noted the need to rightsize the readiness process based on the complexity of the service and time its use early in the project lifecycle. The ability to adapt processes and systems (regardless of content), with availability of “light” versions for busy users, has been previously recognized [54–56].

### Limitations

Although this study yields interesting findings, it is not without limitations. First, this study aimed to explore application of the R = MC^2^ system. As a naturalistic observational study, its utility is not in assessing the effectiveness of the readiness process but rather in gaining early insights into use of a readiness system. Second, both the sample size and the recruitment approach into the CMMA initiative limit generalizability of the findings. This research might be particularly relevant for clinics, health systems, and pharmacies that already demonstrate a certain level of motivation and ability to implement a new service. Finally, the fact that the readiness process overlapped with implementation of the service needs to be considered in the interpretation of the data. It is important to note that engaging simultaneously (rather than sequentially) in planning and implementation is more likely to reflect what actually occurs in busy practices.

## Conclusion

In conclusion, this study provides early insights into the value of a readiness process, R = MC^2^, for healthcare settings implementing a pharmacy care intervention. Readiness should be considered as an essential aspect of any implementation planning process, alongside other preparatory activities such as conducting needs and resources assessments, goal setting, patient engagement, review of available best practice interventions, and development of implementation plans. Future research could build on these findings in a number of ways, including further exploring the optimal readiness process for different needs and contexts; evaluating the impact of readiness building strategies on implementation effectiveness, and ultimately patient outcomes; and further refining the conceptualization of readiness by identifying essential components and defining readiness thresholds for each implementation stage. If readiness is to be integrated into routine practice as part of any implementation effort, it is critical to gain a better understanding of its application, value, and effectiveness.

## Data Availability

The datasets used and/or analyzed during the current study are available from the corresponding author on reasonable request

## Abbreviations

AIMM: Alliance for Integrated medication Management
CMM: Comprehensive medication management
CMMA: Concordia Medication Management Accelerator
CMO: Center for Medication Optimization
CMR: Comprehensive medication review
CUW: Concordia University Wisconsin
Med Sync: Medication synchronization
MTM: Medication therapy management
RMT: Readiness monitoring tool
